# The transcription factor NRSF contributes to epileptogenesis by selective repression of a subset of target genes

**DOI:** 10.7554/eLife.01267

**Published:** 2014-08-12

**Authors:** Shawn McClelland, Gary P Brennan, Celine Dubé, Seeta Rajpara, Shruti Iyer, Cristina Richichi, Christophe Bernard, Tallie Z Baram

**Affiliations:** 1 Department of Anatomy and Neurobiology, University of California, Irvine, Irvine, United States; 2 Department of Pediatrics, University of California, Irvine, Irvine, United States; 3 Laboratoire Epilepsie et Cognition, Institut National de la Santé et de la Recherche Médicale, Marseille, France; 4 Department of Neurology, University of California, Irvine, Irvine, United States; Children's Hospital Los Angeles, United States

**Keywords:** neuron-restrictive silencing factor, epilepsy, gene set enrichment analysis, rat

## Abstract

The mechanisms generating epileptic neuronal networks following insults such as severe seizures are unknown. We have previously shown that interfering with the function of the neuron-restrictive silencer factor (NRSF/REST), an important transcription factor that influences neuronal phenotype, attenuated development of this disorder. In this study, we found that epilepsy-provoking seizures increased the low NRSF levels in mature hippocampus several fold yet surprisingly, provoked repression of only a subset (∼10%) of potential NRSF target genes. Accordingly, the repressed gene-set was rescued when NRSF binding to chromatin was blocked. Unexpectedly, genes selectively repressed by NRSF had mid-range binding frequencies to the repressor, a property that rendered them sensitive to moderate fluctuations of NRSF levels. Genes selectively regulated by NRSF during epileptogenesis coded for ion channels, receptors, and other crucial contributors to neuronal function. Thus, dynamic, selective regulation of NRSF target genes may play a role in influencing neuronal properties in pathological and physiological contexts.

**DOI:** http://dx.doi.org/10.7554/eLife.01267.001

## eLife digest

Epilepsy is a common brain disease that can cause disabling seizures. During a seizure, brain cells send out abnormal signals, which can mean that people having seizures may be unaware of their surroundings and may fall or otherwise injure themselves.

Individuals with epilepsy develop changes in their brain cells and in the circuits that connect these cells together. Some people develop epilepsy because they have mutations in genes. Others develop the condition after an injury or a long seizure, which leads to changes in gene expression and therefore changes to the brain's cells and circuits.

In 2011, researchers found that a protein that normally switches off the expression of certain genes during brain development, but which is almost absent in the adult brain, may run amok after a seizure. The level of this protein—a transcription factor called NRSF—increased in the brains of rats that had been caused to have a seizure. A long provoked seizure caused many of the rats to develop epilepsy. But, if NRSF was blocked after the original seizure, the rats were less likely to have further seizures later on. Now McClelland et al., including several of the researchers involved in the 2011 work, have examined what normally happens to the expression of genes after a seizure and what happens when the NRSF transcription factor is blocked.

McClelland et al. found that only a small subset—about 10%—of the genes that can theoretically be silenced by NRSF are switched off in the brain when this protein's levels increase after a seizure. The increased NRSF levels, unexpectedly, did not affect the genes that bind tightly to this transcription factor. Nor did NRSF affect genes that bind loosely. Instead, the genes that the transcription factor binds to with an intermediate strength were the ones that were switched off. McClelland et al. suggest that this ‘mid-range binding’ to NRSF allows the expression of these genes to be increased or decreased in response to there being more or less NRSF in the cell. Genes that bind tightly to NRSF are likely to already have a lot of NRSF bound and are therefore already switched off; and loosely-binding genes would likely need even more NRSF before they are switched off.

The subset of genes that were switched off by the increased levels of NRSF after a seizure code for a number of proteins that brain cells need to be able to effectively send and receive messages. Blocking the ability of NRSF to bind to these genes and switch them off may help to prevent the brain changes that cause epilepsy.

**DOI:** http://dx.doi.org/10.7554/eLife.01267.002

## Introduction

Epileptic networks arise via numerous mechanisms (Pitkänen et al., 2011; Vezzani et al., 2011; Kim et al., 2012; Hildebrand et al., 2013; Rowley and Patel, 2013). Changes in neuronal properties and resulting alterations of network behavior constitute one of the important mechanisms of epileptogenesis (Yaari and Beck, 2002; Noam et al., 2011; Goldberg and Coulter, 2013). Neuronal properties may change because of genetic mutations in crucial neuronal genes (Meisler and O'Brien, 2012; Oliva et al., 2012; Kingwell, 2013; Papale et al., 2013) or from insult-related alterations of gene expression that affect the levels and function of their products. Whereas altered expression of individual ion channels (Brewster et al., 2002; Khirug et al., 2010; Poolos, 2012; van Loo et al., 2012; Maslarova et al., 2013; Shah et al., 2013), neurotransmitter receptors (Gonzalez et al., 2013; Rojas and Dingledine, 2013), and other cellular components (Zeng et al., 2009; Maroso et al., 2011; Jimenez-Mateos et al., 2012; Liu et al., 2013) have been extensively reported, the nature and mechanisms of the orchestration of these gene expression changes remain unknown: do epilepsy-provoking insults regulate individual genes via distinct cellular pathways? Alternatively, are there clusters of genes that are co-regulated based on specific common properties, and whose altered expression contributes to the generation of ‘epileptic neurons’? (Brooks-Kayal et al., 2009; McClelland et al., 2011b; Kobow and Blümcke, 2012; Roopra et al., 2012; van Loo et al., 2012). These questions are critical for the understanding and future prevention of certain epilepsies.

Gene expression is dynamically regulated throughout life and influences neuronal phenotype and behavior (Bale et al., 2010; Robison and Nestler, 2011; Sweatt, 2013). This dynamic regulation is mediated by transcriptional enhancers and repressors that bind to specific sequences of target genes and modify their expression (Ballas et al., 2005; Kohyama et al., 2008; Hwang et al., 2010). The repressor neuron-restrictive silencer factor (NRSF/ REST) functions by binding a DNA sequence called neuron-restrictive silencer element (NRSE) to mediate long-term, cell-specific gene repression (Schoenherr and Anderson, 1995) via complex interactions with co-repressors such as mSin3 and Co-Rest (Chen et al., 1998; Andres et al., 1999; Naruse et al., 1999; Roopra et al., 2001; Wood et al., 2003; Belyaev et al., 2004; Kuwabara et al., 2004; Mortazavi et al., 2006; Johnson et al., 2007; Abrajano et al., 2009). NRSF is highly expressed in non-neuronal tissues where it represses neuronal genes (Ballas and Mandel, 2005) and has important roles in neurogenesis and neuronal differentation within the brain (Hsieh et al., 2004; Singh et al., 2008; Westbrook et al., 2008; Mandel et al., 2011; Aoki et al., 2012; Ernsberger, 2012; Kok et al., 2012; Rodenas-Ruano et al., 2012).

NRSF levels are low in mature hippocampus where ∼600 expressed genes contain NRSEs and are thus potentially regulated by the repressor. Hippocampal NRSF expression increases several-fold following seizures (Palm et al., 1998; Garriga-Canut et al., 2006; McClelland et al., 2011a) and following other insults that promote epilepsy (Calderone et al., 2003; Noh et al., 2012; Kaneko et al., 2014). We found that blocking NRSF function attenuated the epileptic phenotype induced by severe seizures (McClelland et al., 2011a). However, it is unclear ‘how’ augmented NRSF levels promote selective neuronal phenotypic changes that provoke epilepsy. In this study, we used large-scale transcriptome arrays, qPCR validation, chromatin immunoprecipitation (ChIP), and gene set enrichment analysis (GSEA) to examine the basis of NRSF-dependent gene expression changes during epileptogenesis. Surprisingly, a twofold to threefold increase of NRSF levels, induced by epilepsy-provoking seizures, led to repression of only a subset of potentially regulated genes, and these were rescued by interfering with NRSF function. Unexpectedly, genes found to be regulated by NRSF were characterized by mid-range NRSF-binding probabilities during the naive state. This moderate baseline binding enabled a significant increase of NRSF binding to these genes upon modest increases in NRSF tissue levels. NRSF-regulated hippocampal genes included ion channels, receptors, calcium-related molecules, and other transcription factors that govern neuronal function and plasticity, suggesting that their repression might contribute to the pathogenesis of epilepsy.

## Results

### NRSE-containing genes are preferentially repressed by seizure-provoked increase of hippocampal NRSF levels

**Figure 1.**
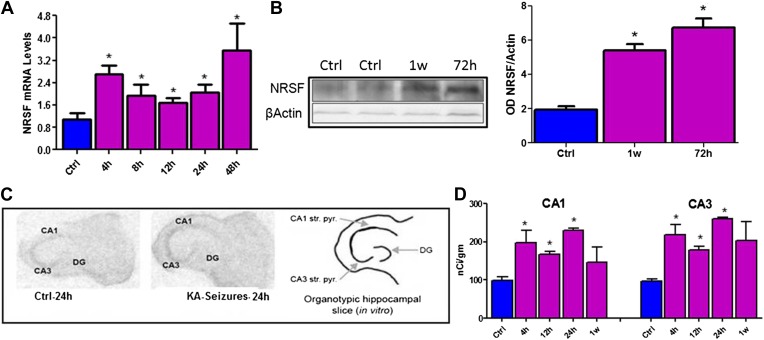
Kainic acid (KA)-seizure-induced increase of NRSF expression. (**A**) Time-course of NRSF mRNA expression levels following KA-induced seizures, n = 4/group. (**B**) Representative western blot image of NRSF protein levels in control (ctrl) animals and animals at 72 hr and 1 week post KA-induced seizures. Quantification of NRSF protein levels using optical density measurements (ctrl 1.96 ± 0.18, n = 6; KA+72hr 6.75 ± 0.54, n = 3; KA+1 week: 5.42 ± 0.36, n = 3). (**C** and **D**) In situ hybridization and quantification of NRSF mRNA in organotypic hippocampal slice cultures which had undergone KA-induced seizure-like events. Quantification of mRNA in pyramidal cell layer was performed in control cultures as well as cultures 4 hr, 12 hr, 24 hr, and 1 week following seizure-like events in CA1 and CA3 region of the hippocampus, n = 4–8/group, *p<0.05. **DOI:** http://dx.doi.org/10.7554/eLife.01267.003

NRSF mRNA and protein expression in mature hippocampus were increased following long seizures induced by systemic administration of the glutamate receptor agonist, kainic acid (KA) (Figure 1A,B). The direct dependence of increased NRSF expression on network activity (seizures) was tested by examining NRSF levels in vitro, using organotypic hippocampal slice cultures, where most potential confounders were excluded. KA provoked seizure-like electrophysiological events in the hippocampal slice (Richichi et al., 2008) and led a to time-dependent increase of NRSF mRNA and protein levels (Figure 1C,D).

**Figure 2.**
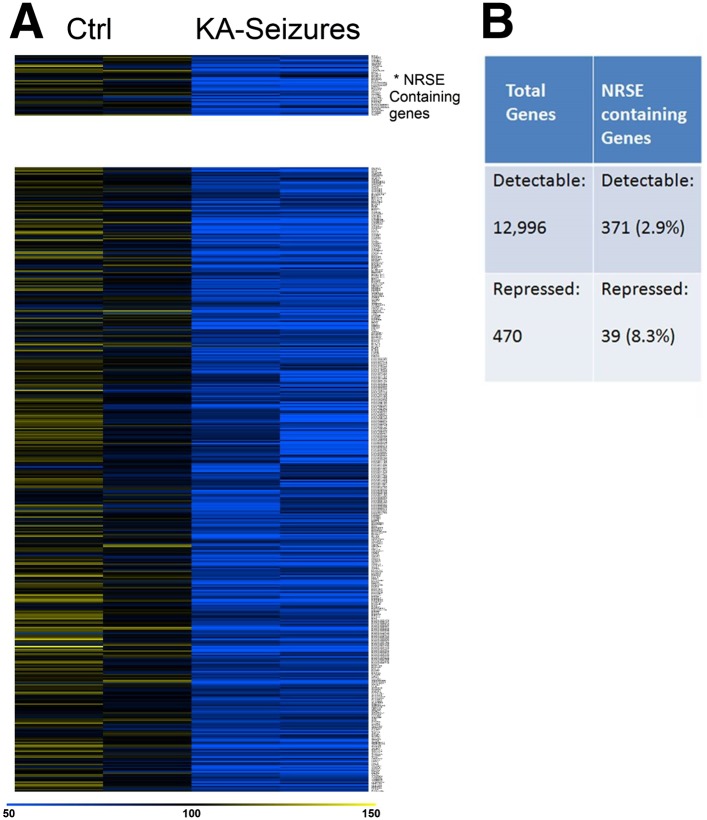
NRSE-containing genes are enriched among hippocampal genes repressed after network activity. (**A**) Heat map representing genes with repressed mRNA expression levels in two representative samples from hippocampi derived from control (Ctrl) and from hippocampi from KA-seizures-experiencing rats. Expression levels are represented by color using a scale from 50% to 150% of expression, where yellow is the highest and blue is the lowest. (**B**) Relative abundance of NRSF target genes among total detected genes vs those repressed by KA-seizures. **DOI:** http://dx.doi.org/10.7554/eLife.01267.004

Because cellular plasticity often derives from large-scale changes in gene expression (Robison and Nestler, 2011), we examined for such large-scale expression changes here using a microarray analysis. We initially screened the microarray data for candidate genes whose expression was repressed following KA-induced seizures and examined whether these genes contained functional NRSEs. Expression of 12,996 genes was detected in the CA1 region of the rat hippocampus of which 371 (2.8%) contained NRSE sites (Johnson et al., 2007). 470 genes whose expression was significantly repressed by KA-induced seizures were identified by this initial candidate gene analysis that employed routine statistical significance (Subramanian et al., 2005; Yukhananov and Kissin, 2008; Borg et al., 2010; Oberbauer et al., 2010; Stark et al., 2010). Of these, 39 (8.3%) contained NRSF binding sites (Figure 2A). The significant enrichment in the fraction of NRSE-containing genes in the repressed vs total gene population (8.3% vs 2.8%) indicated that NRSF-regulated genes were preferentially repressed by epilepsy-provoking network hyperactivity (Figure 2B).

### Only a subset of NRSE-containing genes are regulated by NRSF in mature hippocampus

**Figure 3.**
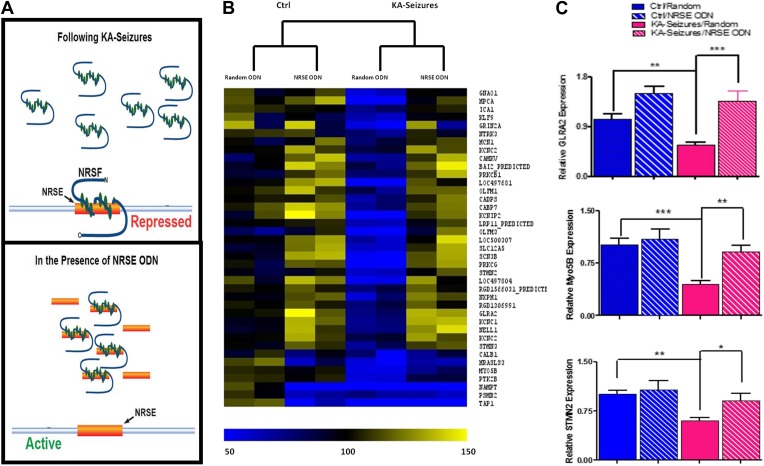
Abrogation of NRSF binding to target genes rescues the majority of NRSE-containing genes repressed by KA-seizures. (**A**) A schematic illustrating the mechanism of action of NRSF following a seizure-induced increase and the decoy oligodeoxynucleotide (ODN) intervention strategy with the expected outcome. (**B**) A heat map representation of the changes in mRNA expression levels of genes that contain a putative NRSE site that were down-regulated 48 hr after KA-induced seizures. Heat map compares representative samples from two hippocampi, from each of four experimental conditions: 'controls' receiving random ODNs (n = 4); ‘controls’ receiving NRSE ODNs (n = 4); ‘KA-seizures’, rats sustaining KA induced seizure activity and receiving random ODNs (n = 3); ‘KA-seizures + NRSE-ODN’, rats sustaining KA-induced seizure activity and receiving NRSE-ODNs (n = 4). Samples and genes are plotted using hierarchical clustering using Euclidean distance and average linkage. Expression level is depicted by color using a scale from 50% to 150% of expression, where yellow is the highest and blue is the lowest. (**C**) Independent analysis of gene expression using qPCR. Several genes that were both repressed by seizure activity and rescued by interference with NRSF function were tested (Glra2, *Myo5B, Stmn2*), and results analyzed using two way ANOVA. *Myo5B* F_(1,18)_ = 9.35, p = 0.007; *Glra2* F_(1,17)_ = 46.89, p = 0.0001; *Stmn2* F_(1,18)_ = 1.97, p = 0.047, n = 4/group. **DOI:** http://dx.doi.org/10.7554/eLife.01267.005

Whereas 371 of the detected hippocampal genes contained an NRSE and were therefore potential NRSF targets, only 39 of these were repressed by KA-induced seizures when NRSF levels increased twofold to threefold. To examine if the correlation between NRSF levels and the repression of this subset (∼10%) of NRSE-containing genes was causal, we employed a decoy oligonucleotide strategy to block NRSF binding to the NRSE sequences of genomic DNA (Akhtar et al., 1991; Szklarczyk and Kaczmarek, 1995; Gilar et al., 1998; Soldati et al., 2011; Sedaghat et al., 2013). We generated oligodeoxynucleotides (ODNs) comprised of the NRSF binding sequence (NRSE), modified their backbone for stability and infused them into the brain. These ODNs acted as ‘decoys’, binding to cellular NRSF and inhibiting its ability to bind to target genes (Figure 3A) (McClelland et al., 2011a).

Consistent with the low baseline levels of NRSF in naive hippocampus, preventing the binding of the repressor to target genes had little effects on the expression of most of the 39 genes (compare “random ODN” and NRSE–ODN columns in the control brain, left side of Figure 3B). Of the 39 NRSE-containing genes that were repressed by seizures in the presence of random ODNs, (depicted in blue in Figure 3B) the majority (28) were no longer significantly repressed when NRSF binding to target genes was inhibited (compare the [KA-seizures + NRSE ODN] group to either of the control groups; Figure 3B). These microarray data supported the idea that in mature hippocampus a twofold to threefold change in NRSF levels influenced the expression of only a subset of putative NRSF target genes and this repression required binding of NRSF to the chromatin. To examine the validity of the microarray data supporting this observation, we performed qPCR on a subset of genes to confirm both their seizure-induced repression as well as their ‘rescue’ by NRSE-ODN administration (Figure 3C).

### Physical binding of NRSF co-varies with tissue levels specifically at genes that are regulated by the repressor

**Figure 4.**
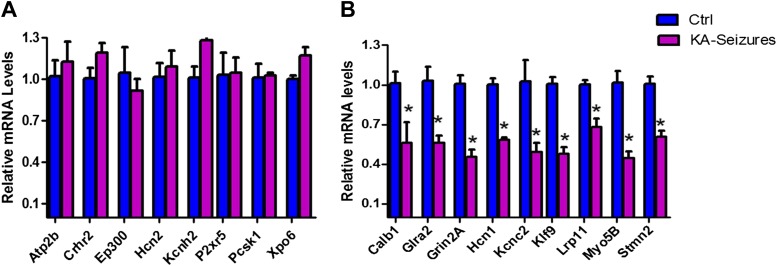
qPCR validation of microarray results. (**A**) A selection of NRSE containing genes (*Atp2b, Crhr2, Ep300, Hcn2, Kcnh2, P2xr5, Pcsk1, Xpo6)* whose expression was unchanged according to the microarray following KA-seizures were measured using qPCR to validate the microarray n = 4/group. (**B**) qPCR measurement of a selection of the NRSE containing genes (*Calb1, Glra2, Grin2a, Hcn1, Kcnc2, Klf9, Lrp11, Myo5b, Stmn2)* whose expression was down-regulated following KA-seizures and rescued by NRSE-ODNs n = 4/group, p*<0.05. **DOI:** http://dx.doi.org/10.7554/eLife.01267.006

We next examined the microarray data by performing qPCR analysis of a subset of NRSE-containing genes that were found to be repressed on the array and a second subset of genes whose expression was not altered on the array following KA-seizures (Figure 4A,B). These qPCR analyses revealed excellent correlation with the microarray and both methods distinguished between the NRSE-containing genes that were or were not repressed by seizures.

**Figure 5.**
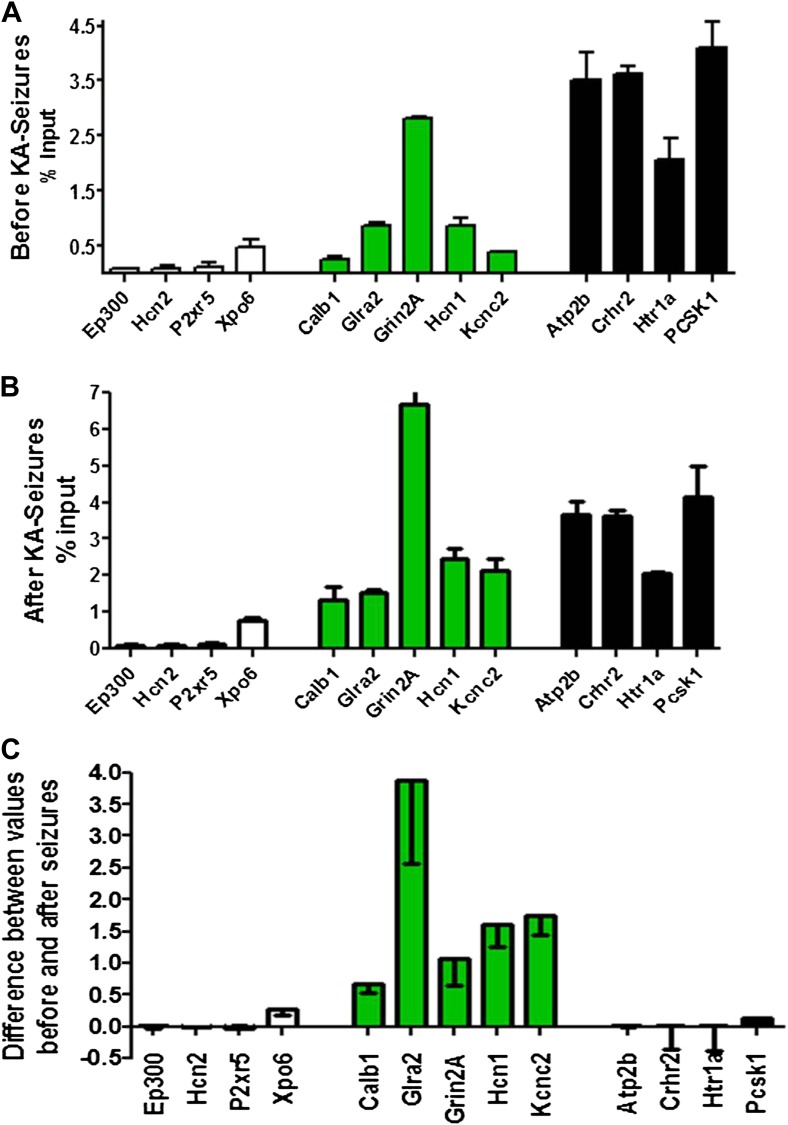
Physical binding of NRSF co-varies with tissue levels specifically at genes that are regulated by the repressor. (**A**) NRSF binding (expressed as percent of input) to selected NRSE-containing genes in naive hippocampus with genes whose expression is repressed by seizure-induced NRSF increase represented in green, genes where NRSF occupancy was low are depicted in white, while genes where NRSF binding was abundant are depicted in black. (**B**) NRSF occupancy (percent input) at the same gene set in the hippocampus 48 hr following KA-induced seizures. (**C**) Graphical depiction of the changes (Delta) in NRSF occupancy at NRSE-containing gene sets comparing occupancy following KA-induced seizures to occupancy in the naive state. Genes whose expression was repressed are represented in green, n = 4–6/group, p*<0.05. **DOI:** http://dx.doi.org/10.7554/eLife.01267.007

To probe the basis of the differential regulation of these two gene groups, we compared the binding of NRSF to the repressed and non-repressed genes in both the naive hippocampus and following KA-seizures. Figure 5A depicts the relative binding of NRSF to 13 genes that are presented in the order of increasing NRSF binding in the naive hippocampus. Genes repressed by the seizures are shown in green. Figure 5B shows the relative binding of NRSF to the same genes in hippocampi taken from rats undergoing seizures. For each of these genes, we calculated the increment in NRSF binding after the seizures (when NRSF levels are increased) relative to the naive state (Figure 5C). As apparent in this figure, (a) genes repressed by seizure-induced increases of NRSF levels were those that had a large increment in NRSF binding. In addition, (b) this increment arose on the background of moderate binding levels in the naive state.

Specifically, the effects of seizure-induced increase of NRSF levels on NRSF binding to the chromatin correlated with the degree of repressor binding in the naive state: binding of NRSF to certain genes (*Ep300, Hcn2, P2xr5, Xpo6*) was modest and did not increase significantly upon seizure-induced increase of NRSF levels (white columns of Figure 5A–C). NRSF binding was very robust at NRSE sites of other genes (*Atp2b, Crhr2, Htr1a, Pcsk1*), and the degree of binding was relatively independent of NRSF tissue levels (black columns of Figure 5A–C). In contrast, seizure-induced increase of NRSF levels markedly augmented NRSF binding to genes such as *Calb1, Glra2, Grin2A, Hcn1, and Kcnc2*, where binding was moderate in the naive hippocampus. The latter set of genes was those preferentially repressed by NRSF in mature hippocampus (Figure 4B and Figure 5C).

### A potential ‘dynamic range’ of repressor binding might enable gene regulation by moderate fluctuations of NRSF levels

**Figure 6.**
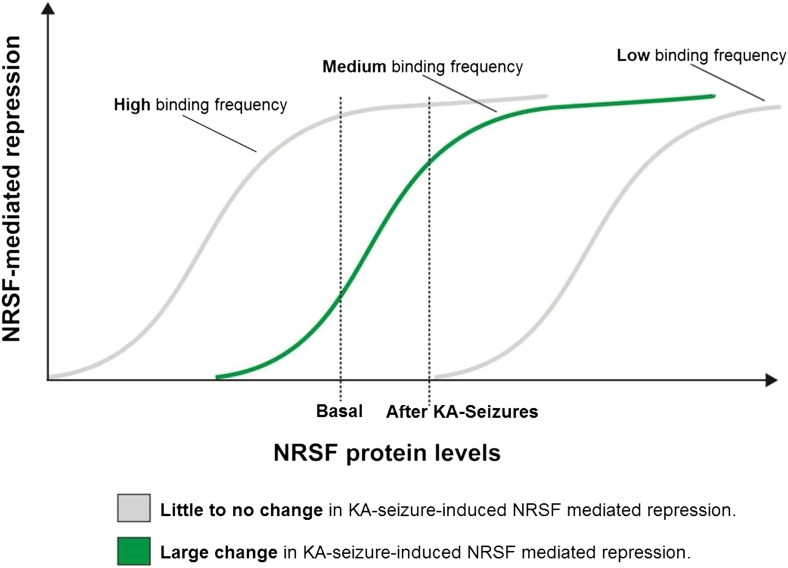
A potential ‘dynamic range’ of repressor binding might enable gene regulation by moderate fluctuations of NRSF levels. A graphical representation of our proposed hypothesis based on our observations that only a subset of NRSE-containing genes are functionally repressed by seizure-induced increases in NRSF levels and that these genes appear to have moderate NRSF binding in the naive brain. **DOI:** http://dx.doi.org/10.7554/eLife.01267.008

As shown above, only a subset of NRSE-containing genes in mature hippocampus was repressed by seizure-dependent increase of hippocampal NRSF levels and ‘rescued’ when NRSF binding was prevented. Interestingly, this sub-population of putative NRSF target genes had moderate repressor binding in the naive state which increased markedly after seizures. This led us to propose a simple model to explain these findings. According to the model (Figure 6), in genes with very low binding to NRSF, a moderate increase of NRSF levels would not alter repressor binding appreciably. Similarly, in genes with high binding frequency to NRSF, NRSEs would likely be occupied even by the low NRSF levels in the naive hippocampus. Increased NRSF levels would be unlikely to augment occupancy or promote suppression of these genes. In contrast, when NRSF binding is ‘mid-range’, then relatively small fluctuation of NRSF levels would result in major changes of repressor binding and gene repression.

**Figure 7.**
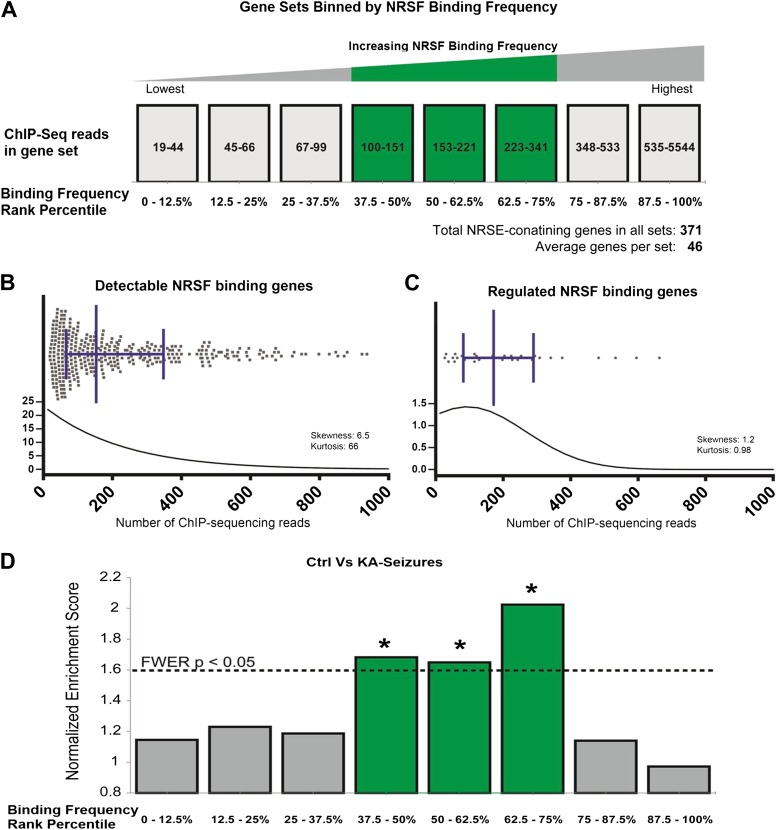
Genes regulated by seizure-dependent changes in NRSF function possess a distinct range of NRSF binding frequencies. (**A**) Diagram illustrating sets of genes that were binned according to binding frequency based on Johnson et al., 2007. Gene sets were numbered in increasing order of their NRSF binding frequencies. Mid-range NRSF-binding frequencies are in green. (**B** and **C**) A binding frequency metric was established for each NRSF-binding gene based on the number of ChIP-Seq reads from published data (Johnson et al., 2007). The distribution of (**B**) all microarray-detectable NRSF-binding genes was compared to the distribution of (**C**) genes regulated by seizure-dependent NRSF changes, that is, those genes significantly repressed by network hyperactivity and rescued by NRSE–ODN treatment. Presented are scatter dot plots, with median with interquartile ranges. Below them are regression fit histogram plots. (**D**) Comparing microarray data from control rats to that of rats experiencing KA-seizures (both with scrambled ODN), using GSEA, illustrates that three gene-sets were significantly enriched in the control rats (repressed in the seizure + scrambled-ODN rats) and these fell in the mid-frequency category (see Table 1 for numeric values). **DOI:** http://dx.doi.org/10.7554/eLife.01267.009

We sought to refute or support this broad supposition by examining available information about binding frequencies of NRSF to a large gene set. A comprehensive analysis of NRSF binding probabilities has been reported, based on ChIP-sequencing data (ChIP-Seq) (Johnson et al., 2007). The published binding frequencies were derived from a cell line, and it is likely that several cell- and tissue-specific parameters influence NRSF binding to the chromatin (Lawinger et al., 2000; Zuccato et al., 2007; Gillies et al., 2011; Lovén et al., 2013; Whyte et al., 2013). Cognizant of these potential caveats, we simply employed the data set to estimate broadly the binding frequencies of NRSE-containing genes to NRSF. We partitioned these putative NRSF target genes into 8 sets (bins) based on their NRSF binding-frequency rank (Figure 7A). We then partitioned (binned) all hippocampal NRSE-containing genes that were detectable in the transcriptome arrays into 8 sets based on their predicted NRSF binding-frequency rank using the Johnson data set.

We compared the binding frequency distribution of all of the hippocampal NRSE-containing genes to that of the subset of genes that were functionally regulated by NRSF (Figure 7B). In the overall population of NRSE-containing hippocampal genes, binding frequencies ranged from 19 to 5544 (mean 279; median 153; standard deviation 429). In contrast, NRSF-binding scores of the genes that were functionally regulated by NRSF ranged from 27 to 663 (mean 214; median 172; standard deviation 166). Whereas the frequency medians of all NRSE-containing hippocampal genes and NRSF-regulated NRSE-containing genes were not significantly different (Mann–Whitney p = 0.86), the scatter plots and regression-fit histograms demonstrated that the gene population regulated by NRSF in the context of epileptogenesis was more tightly clustered and had distinct distribution properties. Skewness was 6.5 in all detected NRSE-containing genes vs 1.2 in the regulated genes; Kurtosis was 66 in all of the genes and 0.98 in the regulated genes. Notably, whereas NRSF-binding distribution of the total NRSE-containing hippocampal gene population was exceedingly different from normal (all: Kolmogorov–Smirnov test p < 0.0001, D'Agostino and Pearson omnibus normality test p < 0.0001), the distribution pattern of the regulated gene population was much closer to normal (Kolmogorov–Smirnov test p > 0.10, D'Agostino and Pearson omnibus normality test p = 0.012). Together, these clustering patterns suggest that the population of NRSF-regulated hippocampal genes is distinct and characterized by a relatively narrow, normally-distributed range of NRSF-binding frequencies.

**Figure 8.**
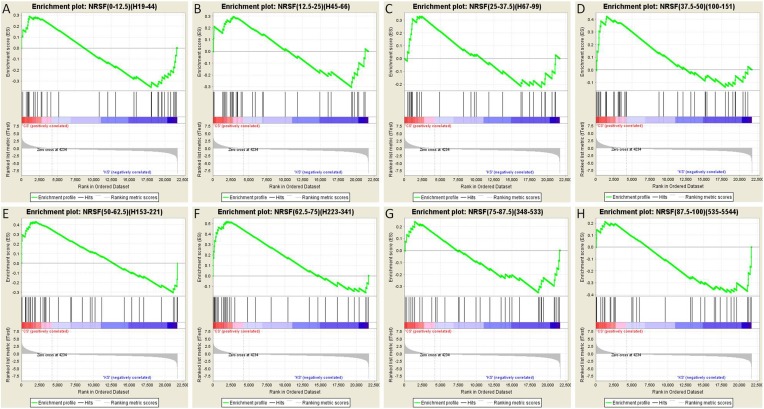
Gene set enrichment analysis (GSEA, Broad Institute, MIT) curves of hippocampal NRSE-containing genes. These genes have been classified (‘binned’) into eight groups by increasing order of their NRSF binding frequency rank percentile. Hence, these genes might be categorized as low-binding frequency (**A**–**C**), mid-binding frequency (**D**–**F**), and high-binding frequency (**G**–**H**). Each graph shows enrichment plots comparing gene expression in KA-seizures vs controls in the presence of random ODNs. Please see Table 1, top, for the numeric values and parameters of the analyses. **DOI:** http://dx.doi.org/10.7554/eLife.01267.010

To further test this idea, we analyzed the array data using gene set enrichment analysis (GSEA, MIT Broad Institute) (Mootha et al., 2003; Subramanian et al., 2005). GSEA is a computational method that determines whether an a priori defined set of genes shows statistically significant, concordant differences between two experimental groups. Comparison of the control group to the seizures group for each binned gene set as described above resulted in a graphic representation depicting the GSEA-normalized Enrichment Scores (NES), obtained from the Broad Institute's molecular signature database (MSigDB), for each set of partitioned NRSE-containing genes (Figure 7D). The GSEA graphs are shown in Figure 8, and the analysis parameters, including false discovery rate [FDR] and family-wise error rates [FWER] are found in Table 1. The expression of three of these ‘bins’ (gene sets partitioned by binding frequency) was significantly enriched in the control group (i.e., repressed in the seizure group) when compared to the levels of enrichment of gene sets composed of random permutations of genes (FWER *p < 0.05). Notably, the three significantly enriched gene-sets were not composed of genes with the highest NRSF binding frequency; rather, they consisted of genes between the 37.5^th^ and 75^th^ binding frequency rank percentile (Figure 7D; Table 1; Figure 8). In addition, when binding-frequency binned GSEA was used to compare the control group to the KA-seizure group receiving the NRSE–ODN treatment (i.e., with blocked NRSF function), the three binned gene sets were no longer significantly different from those in the control group (Table 1).

**Table 1.** Gene-set enrichment analyses focusing on NRSF **DOI:** http://dx.doi.org/10.7554/eLife.01267.011

Gene set name	NES	NOM p-val	FDR q-val	FWER p-val
Ctrl vs Activity (both with scrambled ODN)				
NRSF(0-12.5)(19-44)	1.1449	0.2891	0.3824	0.8710
NRSF(12.5-25)(45-66)	1.2296	0.5170	0.5150	0.9870
NRSF(25-37.5)(67-99)	1.1865	0.2140	0.2020	0.2940
**NRSF(37.5-50)(100-151)**	**1.6817**	**0.0102**	**0.0212**	**0.0160**
**NRSF(50-62.5)(153-221)**	**1.6487**	**0.0258**	**0.0175**	**0.0210**
**NRSF(62.5-75)(223-341)**	**2.0243**	**0.0000**	**0.0000**	**0.0000**
NRSF(75-87.5)(348-533)	1.1396	0.2777	0.5563	0.8600
NRSF(87.5-100)(535-5544)	0.9720	0.1868	0.7621	0.7200
Ctrl vs Activity (both with NRSE-ODN)				
NRSF(0-12.5)(19-44)	1.1485	0.2634	0.3881	0.6590
NRSF(12.5-25)(45-66)	1.1842	0.2000	0.2071	0.6050
NRSF(25-37.5)(67-99)	1.3069	0.1165	0.1323	0.3540
NRSF(37.5-50)(100-151)	1.4666	0.0458	0.0646	0.1450
NRSF(50-62.5)(153-221)	1.4762	0.0270	0.0880	0.1300
NRSF(62.5-75)(223-341)	1.5241	0.0294	0.1276	0.0970
NRSF(75-87.5)(348-533)	1.2320	0.4121	0.4184	0.8400
NRSF(87.5-100)(535-5544)	1.0232	0.1824	0.5207	0.5190

NRSF is significantly enriched in gene sets with moderate binding probability even after false discovery corrections (FDR q-val), when the control and activity groups are compared (top; in bold). When the function of NRSF is blocked, that is, when comparison groups are the control and seizure-activity groups both treated with the NRSE-ODN, NRSF target gene-sets the bins (gene-sets) with moderate binding-frequencies are no longer enriched among differentially expressed genes.

### Genes regulated by NRSF are key molecules that contribute to neuronal properties

To better identify genes regulated by moderate fluctuations of NRSF in mature hippocampus, we followed the screen of microarray data for candidate genes with further analyses to control for false discovery (type I error). This approach uncovered genes that were significantly different in the (seizure + random-ODN) hippocampi vs the other groups (Table 2; p < 0.05, one-way ANOVA, Benjamini–Hochberg FDR p < 0.25). Genes that were both repressed by the seizures and ‘rescued’ by blocking NRSF–NRSE interactions encoded primarily molecules that are well-known to contribute to neuronal function and plasticity. These included ion channels and their accessory subunits, neurotransmitter receptors, genes involved in calcium-mediated cellular cascades, and phospho-enzymes (Table 2). Several of these genes encode for transcription factors (e.g., Kruppel-like factor 9 [*Klf9*]), or proteins involved in signaling cascades that might influence downstream gene expression (*Grin2A*, *PKC*, *Nell1*), suggesting that they might mediate secondary (indirect) effects of NRSF. Notably, this NRSF-regulated gene set is crucial in influencing the function of both individual neurons (Garriga-Canut et al., 2006) and neuronal network behavior. Their importance is supported by the fact that preventing their repression attenuated seizure-provoked neuroplasticity that promoted epilepsy (McClelland et al., 2011a).

**Table 2.** Candidate list of seizure-dependent NRSF regulated genes **DOI:** http://dx.doi.org/10.7554/eLife.01267.012

Ion channels, accessory subunits or receptors				
HCN1	hyperpolarization-activated cyclic nucleotide-gated channel 1 (Hcn1)	*	†	‡
LRP11	low density lipoprotein receptor-related protein 11	*	†	‡
KCNC2	potassium voltage gated channel, Shaw-related subfamily, member 2	*	†	‡
KCNIP2	Kv channel-interacting protein 2 (Kcnip2), transcript variant a	*	†	
BAI2	brain-specific angiogenesis inhibitor 2 (predicted)	*	†	
SCN3B	sodium channel, voltage-gated, type III, beta (Scn3b)	*		
SLC12A5	solute carrier family 12 member 5 (Slc12a5), (KCC2)	*		
GRIN2A	Glutamate receptor, ionotropic, N-methyl D-aspartate 2A (Grin2a)	*		
KCNC1	K voltage gated channel, Shaw-related subfamily, member 1, transcript 2 (Kcnc1)	*		
GLRA2	glycine receptor, alpha 2 (Glra2)	*		
NTRK3	neurotrophic tyrosine kinase, receptor, type 3 (Ntrk3)	*		
Calcium-mediated cellular cascades				
CADPS	Ca++-dependent secretion activator (Cadps)	*	†	‡
CALB1	calbindin 1 (Calb1)	*	†	
HPCA	hippocalcin (Hpca)	*	†	
CABP7	calcium binding protein 7 (Cabp7)	*	†	
MYO5B	myosin Vb (Myo5b)	*		
CAMKV	CaM kinase-like vesicle-associated (Camkv)	*		
Phosphoenzymes				
GNAO1	guanine nucleotide binding protein, alpha O (Gnao1)	*	†	‡
PRKCG	protein kinase C, gamma (Prkcg)	*		
PRKCB1	protein kinase C, beta 1 (Prkcb1)	*		
NELL1	NEL-like 1 (chicken) (Nell1)	*		
Others				
KLF9	Kruppel-like factor 9 (Klf9)	*	†	‡
STMN2	stathmin-like 2 (Stmn2)	*	†	
OLFM3	olfactomedin 3 (Olfm3)	*	†	
ICA1	islet cell autoantigen 1 (Ica1)	*	†	
STMN3	stathmin-like 3 (Stmn3)	*		
OLFM1	olfactomedin 1 (Olfm1)	*		
NXPH1	neurexophilin 1 (Nxph1)	*		

Genes were designated as ‘NRSE-containing’ based on NRSF ChIP-sequencing data from previous studies (Johnson et al., 2007). For inclusion in the initial list of candidate genes for NRSF regulation, genes were considered significantly repressed if their expression was reduced by more than 20% after KA-induced seizures (p < 0.05). Genes considered ‘rescued’ were significantly repressed after seizures but not significantly repressed after ODN infusions [seizures + NRSE-ODN treatment]. This candidate gene approach was followed by measures to correct for FDR (see ‘Materials and methods’).

* genes (28) with expression reduced by > 20% after KA-induced activity, and rescued by NRSF ODN; p < 0.05.

† genes (14) found with ANOVA, Benjamini–Hochberg FDR <0.25.

‡ genes (6) found with one-way ANOVA, unadjusted p value <0.05.

## Discussion

The key findings of these studies are: (1) NRSF contributes to epileptogenesis by regulating a group of genes that critically influence neuronal function; (2) whereas hundreds of hippocampal genes are potential NRSF targets because they contain NRSEs, only a few dozen are actually repressed by the transcription factor in the context of seizure-induced epilepsy as evident from their rescue when the function of NRSF is blocked; (3) the basis of the selective regulation of a subset of NRSE-containing genes includes the mid-range binding frequencies of these genes to the repressor, so that moderate fluctuations in NRSF levels influence the degree of their binding to the repressor. Whereas these findings were observed in the context of epileptogenesis, a neuroplasticity that leads to disease, they may apply to a broad spectrum of physiological and pathological activity-dependent plasticities in the mature hippocampus.

NRSF is a transcription factor with protean effects. Whereas much is known about the role of this molecule in non-neuronal tissues and in neuronal differentiation, much less information is available about the function of NRSF in mature brain (Korosi et al., 2010; McClelland et al., 2011b). More recently, a protective role of NRSF has been proposed in aging and dementia (Lu et al., 2014). In addition, NRSF may be involved in neurological disorders including Huntington disease (Zuccato et al., 2007; Soldati et al., 2011) and stroke (Calderone et al., 2003; Kaneko et al., 2014).

The contribution of NRSF to epilepsy and its development (epileptogenesis) is currently the focus of intense study (Roopra et al., 2012; Goldberg and Coulter 2013; Pozzi et al., 2013). We previously found that interfering with NRSF binding to the chromatin-attenuated epileptogenesis. Interestingly, Hu et al. found that the conditional deletion of NRSF in the forebrain resulted in increased susceptibility to kindling and enhanced mossy fiber sprouting (Hu et al., 2011). The apparent discrepancy between these studies is likely a result of the significantly different approaches. Complete deletion of NRSF in ‘naive’ neurons may induce large cascades of transcriptional changes rendering neurons more susceptible to seizures. In contrast, acute disruption of NRSF function ‘after’ an epilepsy-provoking insult, as performed in our current and previous studies, provides a more direct approach to elucidate the epileptogenic functions of NRSF because it does not interfere with NRSF function prior to the epilepsy-inducing insult. This point is important also because the role of NRSF in mature neurons is not fully understood, as discussed above.

In previous studies, we focused on the rescue of a single gene coding for the ion channel HCN1 (McClelland et al., 2011a). However, whereas it was clear that NRSF likely regulated numerous additional genes that are involved in the epileptogenic process, the identity of these genes was unclear. In this study, we used large-scale transcriptome arrays and found that NRSE-containing genes were enriched among seizure-suppressed genes and that the majority of these genes were restored to control levels after treatment with decoy NRSE–ODNs. The fact that preventing the repression of (‘rescuing’) these genes led to attenuation of epilepsy strongly supports a role for these genes in the transformation of normal neurons (and neuronal networks) into epileptic ones.

Investigating the identity of these genes, we were pleased to find that the majority were already known to contribute to crucial neuronal properties, and many had been previously implicated in pathological states including epilepsy (Sloviter, 1989; Palm et al., 1999; Pathak et al., 2007; Zuccato et al., 2007; van Gassen et al., 2009; Khirug et al., 2010; Soldati et al., 2011; McClelland et al., 2011a; Nikitidou et al., 2012; Kingwell, 2013). This was not surprising, because these genes included ion channels, neurotransmitter receptors, calcium-dependent effectors, and phosphoenzymes (Pernhorst et al., 2011; Dingledine, 2012; Rodenas-Ruano et al., 2012; Maslarova et al., 2013).

Whereas NRSF-mediated repression might theoretically involve hundreds of hippocampal NRSE-containing genes, it was intriguing to discover that in fact only a small subset of detected NRSE-containing genes were repressed by seizures. Most were rescued by blocking NRSF function—as evident using a differential array that included control and insult groups as well as groups where NRSF function was blocked—thus ascertaining that genes that are repressed during epileptogenesis are indeed repressed by the augmented levels of endogenous NRSF. To our knowledge this is the first study demonstrating the specificity of NRSF-mediated gene regulation in mature hippocampus in vivo.

What might be the basis for the apparent selectivity of NRSF repression of only a minority of putative target genes? The binding of NRSF to a gene is required (though not always sufficient; [Belyaev et al., 2004]) for its repression. A number of mechanisms may influence both the degree of NRSF binding as well as the degree of its repressive effect on genes. These include the specific NRSE sequence (Mortazavi et al., 2006; Johnson et al., 2007) that is unlikely to be the cause in the current studies (Supplementary file 1A). Other important mechanisms include the interaction of NRSF with co-factors (Andres et al., 1999; Naruse et al., 1999; Wood et al., 2003; Gao et al., 2011), as well as additional, still enigmatic effects of the chromatin environment of individual genes (Belyaev et al., 2004), which might be influenced by ‘super-enhancers’ (Lovén et al., 2013; Whyte et al., 2013).

Here, we examined the binding of NRSF to hippocampal genes before and after an epilepsy-provoking insult and found significant increment in genes that were repressed by the factor. This suggested a correlation between repressor occupancy and gene repression (Hammar et al., 2014). To our surprise, basal binding of NRSF in regulated hippocampal genes was moderate. By contrast, most non-regulated genes had either high or low NRSF occupancy in naive hippocampus, with little change after an insult that augmented NRSF levels twofold to threefold. We examined our finding in mature hippocampus by comparing the binding-frequency distribution found in hippocampus to a larger set of NRSE target genes as determined by ChIP-Seq analysis (Johnson et al., 2007). Whereas many factors, as discussed above, may lead to different NRSF binding in cell lines and hippocampus, it was encouraging to find that our estimates for the relative mid-range binding of regulated genes among those expressed in hippocampus held when superimposed on a large NRSE-containing gene set (Johnson et al., 2007). Traditionally, the focus of studies on gene–repressor interaction has been on genes with the strongest binding probability (Nichols et al., 1992; Chen et al., 1998; Singh et al., 2002). However, the genes with the strongest binding for NRSF would likely be constitutively bound to—and suppressed by—NRSF. These genes are unlikely to respond to moderate changes in NRSF levels. Rather, relatively modest seizure-induced changes in the tissue levels of NRSF regulate a subset of NRSE-containing genes that fall within a moderate ‘dynamic range’ of NRSF binding probabilities. This concept may be important for other forms of insult-induced plasticity in the mature brain including ischemia (Noh et al., 2012; Kaneko et al., 2014), peripheral nerve injury (Uchida et al., 2010), and seizure-induced epilepsy (McClelland et al., 2011a; Goldberg and Coulter, 2013) where changes in NRSF levels are moderate.

These findings support a role of NRSF in mature brain that is distinct from its role in development, when NRSF contributes to neuronal identity by repressing neuronal genes in non-neuronal tissues (Schoenherr and Anderson, 1995; Coulson, 2005), and specifies neuronal fate (Kuwabara et al., 2005). In that context, large changes in the expression of NRSF take place: NRSF levels plunge during neuronal differentiation (Gates et al., 2010; Ernsberger, 2012), de-repressing genes that contribute to neuronal identity (Wood et al., 2003; Belyaev et al., 2004; Abrajano et al., 2009). Remarkably, the gene set regulated by moderate changes in NRSF expression in the current study consisted of molecules which are extremely important for neuronal function and plasticity not only during development (Gates et al., 2010; Juliandi et al., 2010; Rodenas-Ruano et al., 2012; Pernhorst et al., 2013), but also in the adult brain (Lepagnol-Bestel et al., 2009; Gao et al., 2011; Pernhorst et al., 2011).

In summary, insult-induced increases of NRSF levels and activity contribute to epileptogenesis via NRSF-mediated repression of a group of genes that critically influence neuronal function. Of the hundreds of NRSE-containing hippocampal genes only a subset are repressed by the transcription factor in the context of seizure-induced epilepsy as evident from their rescue when the function of NRSF is blocked. The basis of the selective regulation of a subset of NRSE-containing genes includes their mid-range binding frequencies to NRSF, so that moderate fluctuations in NRSF levels influence the degree of their binding. These findings inform us about the mechanisms of neuroplasticity in the mature hippocampus.

## Materials and methods

All experiments were performed according to NIH guidelines and approved by the UCI Institutional Animal Care and Use Committee.

### Organotypic slice cultures

Organotypic hippocampal slice cultures were prepared and maintained using the interface technique (Stoppini et al., 1991). Briefly, hippocampi from P8 rat pups of both genders were resected and cut into 400-µm slices using a McIlwain tissue chopper (The Mickle Laboratory Engineering Co. Ltd, Surrey, UK). The slices were collected in ice-cold preparation buffer (100% Minimal Essential Medium, containing 30 mM glucose, and 3 mM glutamine, pH 7.3; Life Technologies, Rockville, MD), then placed onto moistened membrane inserts (Millicell-CM, 30 mm, 0.4 µm pore diameter; Millipore, Bedford, MA), transferred to sterile six-well plates filled with 1 ml culture medium (50% Minimal Essential Medium, 25% Hank's balanced salt solution, 20% heat-inactivated horse serum, 30 mM HEPES, 30 mM glucose, 3 mM glutamine, 0.5 mM ascorbic acid, 1 mg/ml insulin, 5 mM NaHCO_3_, pH 7.3) and incubated in a humidified CO_2_-enriched atmosphere at 36°C. Pairs of adjacent slices (sister cultures) were always compared (i.e., adjacent slices assigned to control and experimental conditions, n = 4–6/group for each time-point). Seizure-like activity was induced after 3 days in vitro by incubating cultures for 3 hr in a medium containing KA (6 µM; Sigma, St. Louis, MO). This seizure-like activity was halted by changing the medium after 3 hr (Richichi et al., 2008).

### Surgery, induction of KA-seizures, and oligonucleotide (ODN) infusion in vivo

Male Sprague–Dawley rats (n = 36) were anaesthetized with inhalation of 4% isoflurane. The rats were shaved and placed in a stereotaxic frame, and their eyes were protected and hydrated with Ocry-gel. A bolus of 2% Lidocaine was injected subcutaneously prior to a midsagittal incision. The skull was exposed and cleaned of blood and periost. Bilateral infusion cannulae were positioned on the cortical surface (−1.0 mm posterior, ±1.5 mm lateral from Bregma) directly above the lateral ventricles, using the coordinates of Paxinos and Watson (Paxinos et al., 1985). The incision was then sutured closed as necessary. All rats received ∼5 ml of 0.9% saline I.P. to rehydrate and aid in recovery from surgery. Rats were allowed 7 days post-surgical recovery before any further experimental procedures were conducted. Status epilepticus (SE) was induced as previously described (McClelland et al., 2011a). Controls received saline. Seizures were observed and scored using the Racine scale (Racine, 1972). One day later, rats were anaesthetized with inhalation of 4% isoflurane. An infusion needle was lowered through the guide cannulae until it reached the cortical surface. The needle was lowered an additional 3.0 mm so the tip was within the lateral ventricle. On the first day, rats received an infusion of 2.0 nmol/µl of ordered NRSE or random ODNs at a volume of 5 µl and a rate of 0.1 µl/min (NRSE: GGA GCT GTC CAC AGT TCT GAA; Random: AGG TCG TAC GTT AAT CGT CGC). The needle remained in place for an additional 10 min to allow for thorough diffusion of the solutions. On the following day, the procedure was identical except that each rat received a volume of 2.5 µl. This infusion protocol was chosen to ensure that an adequate level of ODNs remained in the brain over the course of the experiment (Bristol and Rothstein, 1998).

### In situ hybridization (ISH)

Organotypic hippocampal slice cultures were used at different time points after treatment with KA, and rats receiving KA–SE were decapitated at different time points. Brains were dissected and placed on powdered dry ice. Semi-quantitative analysis of NRSF mRNA levels were accomplished using in situ hybridization (ISH) (Brewster et al., 2002; McClelland et al., 2011a) using S^35^-cRNA probes and slide-mounted frozen sections (20 µm) with the most stringent wash at 0.03 SSC, at 62°C for 60 min. Sections were then dehydrated and apposed to Kodak BioMax film. Exposure time was monitored using 14C standards to maintain the signal in the linear range. Analyses compared control and KA-treated sister cultures derived from the same hippocampus at every time point (n = 4–8 per group per time point) and were performed without the knowledge of treatment group.

### Western blot protein analysis

Animals were decapitated and the hippocampi were rapidly dissected. Tissues were placed in pre-chilled microcentrifuge tubes and processed immediately. The tissue was homogenized in glass/Teflon homogenizers in a cold solution containing 0.32 M sucrose, 0.01 M Tris–HCl (pH 7.4), and Protease Inhibitor Cocktail (PIC Complete; di; Roche, Alameda, CA). Samples were then centrifuged at 800×*g* for 10 min at 4°C, and the pellet was retained for processing to obtain a nuclear-enriched fraction. The supernatant was centrifuged at 16,000×*g* for 40 min at 4°C, and the pellet containing membrane fractions resuspended in RIPA buffer (50 mM Tris–HCl, pH 7.4, 1% NP-40, 1% Triton X-100, 1 mM EDTA, 150 mM NaCl, 1X PIC).

Pellets retained for the nuclear-enriched fraction described above were resuspended in 1 ml hypotonic buffer (10 mM KCl, 10 mM Tris pH 8, 1.5 mM MgCl_2_). Samples were then incubated on ice for 15 min, followed by the addition of 100 μl of 10% NP-40, samples were then spun for 1 min at 16,000×g. The supernatant was then discarded, and 360 μl of RIPA buffer was added to the remaining nuclear pellet. Samples were then sonicated for 10 min on the highest setting (30 s on/30 s off) using a Bioruptor sonicator (Diagenode, Sparta, NJ, USA), to disrupt nuclei. Protein concentration was determined using Bio-Rad Protein assay (Bio-Rad, Hercules, CA).

Protein samples (30 μg) were suspended in Laemmli buffer, separated by 4–12% SDS-PAGE (Lonza, Rockland, ME, USA), transferred to PVDF membrane and blocked in 5% milk for 2 hr at room temperature (RT). Membranes were probed with anti-Actin (1:10,000; Sigma), anti-NRSF (1:2000, sc-25398X; Santa Cruz Biotechnology, Santa Cruz, CA, USA), in PBS with 5% non-fat milk. Membranes were then incubated with secondary IgG-horseradish peroxidase conjugates (1:10,000; AmerCtrl Pharmacia Biotech, Piscataway, NJ, USA) for 1 hr at room temperature. After final washes in PBS-T (6 × 5 min), membranes were incubated with ECL-Plus (Pierce Scientific, Rockford, IL, USA) for 5 min, and immunoreactive bands were visualized by apposing membrane to hyperfilmTM ECL.

A series of ECL exposures were carried out to ensure that non-saturated bands were used for quantification. Western blot data acquisition and analysis were accomplished by measuring the pixel density and area of immunoreactive bands from the ECL films using the ImageJ software (NIH). Values were normalized to actin. Significance level for unpaired t-tests was set at 0.05, and data are presented as mean with standard errors.

### Transcriptome gene array

Hippocampal CA1 regions were dissected using instruments bathed in RNALater (Ambion, Life Technologies, Rockford, IL, USA). The CA1 region was obtained 48 hr after SE from rats of the following groups: ctrl + random ODN (n = 4), ctrl + NRSE–ODN (n = 5), KA-seizures + NRSE–ODN (n = 4), and KA-seizures + random ODN (n = 3). Samples were processed for RNA extraction by SABiosciences using the Array Grade total RNA isolation kit. RNA from an individual rat was applied to separate Illumina RatRef-12. Expression BeadChips for transcriptome analysis of over 22,000 genes. GenomeStudio software was used by SABiosciences (an Illumina CSPro certified service provider) to perform quality control, quantile normalization and background correction, and to execute two-tailed Student's t-tests on the microarray expression results for all genes.

The microarray data complied with ‘minimum information about a microarray experiment’ (MIAME), and the raw data have been deposited in the Gene Expression Omnibus (GEO) (http://www.ncbi.nlm.nih.gov/geo) as GSE 22899.

### qRT-PCR

The CA1 region was micro-dissected from dorsal rat hippocampus or whole hippocampus was dissected using pre-chilled RNase free instruments under a light microscope. Dissected tissue was placed immediately into pre-chilled sterile centrifuge tubes on powdered dry ice and stored at −80°C until use. Total RNA was extracted from CA1 tissue using the RNeasy kit (Qiagen, Sussex, UK) per protocol and quantified using a nanodrop (Thermo Scientific). RNA purity was determined. Double-stranded cDNA was synthesized from total RNA using Roche 1^st^ strand cDNA synthesis kit (Cat # 04379012001; Roche) utilizing random hexamer primers. PCR analysis was performed using cDNA samples in triplicate on a Roche Lightcycler 96 system (Roche). Samples were normalized to Gapdh and relative quantitative amounts were analyzed using the cycle threshold method (2^-ΔΔCt). Minus-reverse transcription and non-template controls were routinely used to eliminate the possibility of genomic contamination or false positive analyses. Primers are provided in Supplementary file 1B.

### Chromatin immunoprecipitation

Whole hippocampal tissue was homogenized in 1% formaldehyde (Sigma) and incubated at room temperature (RT) for 20 min. Samples were centrifuged and the supernatant was discarded. Samples were then subjected to a number of incubations in glycine (Sigma) and hypotonic buffers, all containing a protease inhibitor cocktail (Roche). Samples were then transferred to a Dounce homogenizer and further homogenized before the addition of 10% NP-40 (MP Biochemicals, Solon, OH, USA), then centrifuged and the supernatant was once more discarded. The pellet was dissolved in RIPA buffer with PIC, vortexed, and sonicated (Diagenode) to shear DNA. Samples were then centrifuged for 45 min at 15,000 rpm, and the supernatant was collected and added to tubes containing 1 ml of magnetic DynaBeads (Life Technologies) pre-incubated with 5 µg NRSF antibody/sample or IgG control for 24 hr. Beads and DNA were left to incubate for 1 hr at 4°C. The beads were then accumulated using a magnet and the liquid was aspirated. The beads were washed 5 times with RIPA to remove loosely or non-specifically bound DNA. DNA and beads were separated by adding a Chelex solution (Bio-Rad) and heating samples to 100°C for 10 min. Samples were then centrifuged at 15,000 rpm, and the DNA containing supernatant was transferred to a new PCR tube and stored at 4°C, then quantified using qPCR. NRSF occupancy at NRSE sites was normalized to IgG binding to the DNA and calculated as a percentage of total input. Primers are provided in Supplementary file 1C.

### Transcriptome array analyses

Gene set enrichment analysis (GSEA version 2.5; Broad Institute, Cambridge, MA) (Mootha et al., 2003; Subramanian et al., 2005) was employed to compare the KA-seizures + NRSE–ODN and KA-seizures + random ODN groups to the ctrl + random ODN and ctrl + NRSE–ODN groups using the entire array data set (see Table 1 and Figure 8). The differential expression for each gene was calculated using GSEA software's Signal2Noise metric, which is based on the difference in the mean of the two groups divided by the sum of the two standard deviations. All genes were then ranked and an enrichment score (ES) for each gene set was generated for different gene set collections in the Broad Institute's Molecular Signatures Database (MSigDB—a collection of annotated gene sets for use with GSEA software). The ES was adjusted based on the size of each gene set to obtain the normalized enrichment score (NES) which was used to rank the gene sets according to their over-representation in one group when compared to another. For each gene set the false discovery rate was calculated to determine the probability that its NES represented a false positive finding. The more conservative family-wise error rate (FWER) statistic was also generated. Subsets of NRSF-binding genes from ChIP-Sequencing data previously obtained were populated according to binding affinity rank (Johnson et al., 2007). GSEA was run to determine if any specific subset was significantly contributing to the enrichment and the NES of each subset was plotted.

Statistical analyses of the microarray were stepwise (see Table 2). An initial discovery phase employed direct p values, and this was followed by False discovery rate (FDR) adjustments. This step-wise approach enabled initial candidate gene discovery by decreasing Type II error (and clearly increasing the initial possibility of Type I error). This was important in the context of the moderate seizure-dependent changes of NRSF expression in mature hippocampus where amplified Type II error may result in early rejection of biologically important candidate genes.

Benjamini–Hochberg FDR was employed in subsequent ANOVAs. Genes were considered significantly repressed if they had a greater than 20% reduction in expression at a p-value less than 0.05. Genes were designated as ‘NRSE-containing’ based on NRSF ChIP-sequencing data from previous studies (Johnson et al., 2007). One-way ANOVA was performed using MeV 4.7 (MultiExperiment Viewer from the TM4 Microarray Software Suite) (Saeed et al., 2003, 2006), and post hoc Benjamini–Hochberg FDRadjustments were made to the results using Excel (Microsoft). To avoid Type I error in the context of moderate changes of gene repression in mature hippocampus (see above), we set the FDR at 0.25. To avoid spurious discoveries (Type II error), we validated the repression of a sample of these genes using an independent method (qPCR). Heat maps were generated using MeV 4.7.

### Statistical analysis of qRT-PCR and ChIP

qPCR data were analyzed using a two-way ANOVA to answer the following questions; (a) did ODN treatment have the same effect at all values of status, that is was there any interaction? (b) did the NRSE ODN affect the result? (c) did the long seizures affect the result? ChIP data were analyzed by normalizing NRSF immunoprecipitation to IgG and calculating as a percentage of total input. Control and KA-treated groups were then compared using standard t-test.

## Additional files

Supplementary file 1. (**A**) NRSE sequences for NRSF regulated and non-regulated genes. (**B**) Primers used in qPCR. (**C**) Primers used in ChIP. **DOI:** http://dx.doi.org/10.7554/eLife.01267.013
