# Supplementary material for: The transcription factor NRSF contributes to epileptogenesis by selective repression of a subset of target genes
Source: eLife. 2014 Aug 12;3:e01267. doi: 10.7554/eLife.01267 (PMC4129437; doi:10.7554/eLife.01267)
Supplement: Supplementary file 1. — (A) NRSE sequences for NRSF regulated and non-regulated genes. (B) Primers used in qPCR. (C) Primers used in ChIP. DOI: http://dx.doi.org/10.7554/eLife.01267.013 [file elife01267s001.docx]

Supplemental Materials:

1. Supplementary File 1A: NRSE sequences for NRSF regulated and non-regulated genes.

2. Supplementary File 1B: Primers used in qPCR.

3. Supplementary File 1C: Primers used in ChIP.

**Supplementary File 1A:: NRSE Sequences**

| **Gene** | **NRSE Seq** | **Threshold score** |
| --- | --- | --- |
| *Atp2b* | CCCAGGACCACCAACTGAGAC | 66.4 |
| *Calb1* | AGCAGCACCGCGGACAGCGCC | 91 |
| *Crhr2* | CTGG CCGAAGAGCT GCTTTT | 71 |
| *Ep300* | ATTCAGGACCTCTGGAAGAGC | 71.6 |
| *Glra2* | CACCACCACCACCACCAC | 67.8 |
| *Grin2a* | CGCCCCCCGTGGGGGTGATG | 68.2 |
| *Hcn1* | CTGTCCACAGTTCTGAATCA | 80 |
| *Hcn2* | TCTGCAGCCGCGACAGCTCCT | 69.4 |
| *Htr1a* | GCTGTCCCGGGTGCTGAACC | 85.9 |
| *Kcnc2* | CCTCAGCACCCAGAGAAGCGG | 72.4 |
| *P2xr5* | TCTACCGGGTGCTGCAGCTC | 75.5 |
| *Pcsk1* | TCAGACCTCGGTGCTGAATAT | 78.5 |
| *Xpo6* | GTGTTCCTTGTGCAGCAAGTC | 68.8 |

**Supplementary File 1A::** Listing of NRSE containing genes for which ChIP was performed. NRSE sequences and threshold scores do not correlate with binding / occupancy before or after seizures. Threshold score as determined by Heinemeyer et al., 1998.

**Supplementary File 1B::** Primer sets for qPCR analysis

| **Gene** | **Forward Primer** | **Reverse Primer** |
| --- | --- | --- |
| *Atp2b* | **TAAGGCGACAACCCTCCAT** | **CATGTGTAGGGGTAGAAACATTTG** |
| *Calb1* | **GAAGGAGCTGCAGAACTTGA** | **TCATCTCAGGTGAGCTCCAA** |
| *Crhr2* | **TGAACCCATTTTGGATGACA** | **GTTGATGATGAGGGCGATTC** |
| *Ep300* | **GTTGCTGTTGGTGTTTGTGG** | **CACTCGCTCAATTCTCACA** |
| *Gapdh* | **ATGCCATCACTGCCACTCAGA** | **ACCAGTGGATGCAGGGATGAT** |
| *Glra2* | **ATGCAAGAATCAGGCCAAA** | **CTGTTGATAAAAATATTGCAAGTAACG** |
| *Grin2A* | **CTGGAAGAGGCAGATTGACC** | **CGTAAGCCACAGTGTCTCCA** |
| *Hcn1* | **TCATCCAGCATGGTGTGG** | **GCCGTCTGTCAACTTCATTTC** |
| *Hcn2* | **CACCCCTACAGCGACTTCAG** | **TTTCCCACCATGAACAACAG** |
| *Kcnc2* | **AAGAGTTCCCGCATTCTCTG** | **TGGCAAAGCCCATTTCAG** |
| *Kcnh2* | **TGGGGCTGTCATCATGTTT** | **GCGACCCTACCATGTCCTT** |
| *Klf9* | **TCAGGACCAGAGTGCTTCAG** | **AAATCTCAAAAGTTAACGTGA** |
| *Lrp11* | **TTCTCATGAGCTCCACTTTCC** | **ATTCACCTAAGGTCCCTTCTCA** |
| *Myo5b* | **AGGATGCCCTACAGCAGGT** | **GCACCTGCAGCTTCTTCC** |
| *P2xr5* | **CATCATCCCCACAGTCATCA** | **AGTACCAGGTCGCAGAAGAAAG** |
| *Pcsk1* | **TCCAAAGTTGGAGGCATAAGA** | **AACTAGCCTCAATGGCATCAG** |
| *Stmn2* | **ACCTCCACATTTCTCTGCTCA** | **TTAGCCTGGCTTTGTGGTTT** |
| *Xpo6* | **AGTGTCTGGCCCACCTCTT** | **GAGGAGGGATGGGTGAT** |

**Supplementary File 1C::** Primer sets for ChIP analysis.

| **Gene** | **Forward Primer** | **Reverse Primer** |
| --- | --- | --- |
| *Atp2b* | **ACAGAAAGGTGTGGGAAGGC** | **CTCCTACACCATCCTTGGGC** |
| *Calb1* | **CCGGCGGGATAAATACTGAGAA** | **GCGGCTAGTTTGAGAGAGCG** |
| *Crhr2* | **GTGATCCGATTGAGAGCGGC** | **CACTCACGGGTCGTGTTGT** |
| *Ep300* | **CCTCGTGGTCCTTTCCATCC** | **ATCCATGTAGTAGCCCAGAGC** |
| *Glra2* | **TGCTATGAGTACTGGGAAAACCC** | **AGGCATTAATTACAAGTGCAAAGA** |
| *Grin2a* | **GGTGGAGGTTCCCACTAAGC** | **AAGGTGGAGGATGCAATGGG** |
| *Hcn1* | **CGGTAAACTGCAAGCAAACA** | **AGATGGACGGTCATCAAACC** |
| *Hcn2* | **GGCCTCAGTTTCCCTCATCC** | **CATCCGCAGGGAGAACTTGT** |
| *Htr1a* | **AGATGGCGCTCTGAAGCAAT** | **TGGACTTTGGACTTCTGCCTC** |
| *Kcnc2* | **AGGAACCATTCATTGACTGTACTA** | **TGGATCTCCTTGGGAAGAGGA** |
| *P2xr5* | **GAAGGGGTTTGTGCTGTCTCT** | **GACTCTTGAGGACCCACTCCT** |
| *Pcsk1* | **CAGTCCGGTGAACAAGATGC** | **GGAAGAGAGGGGCAAGTTTCA** |
| *Xpo6* | **ATGGTCTTGGAGGAGGTC** | **AAACAGGAGGCAGAGAGGAAC** |

**Supplemental references:**

Heinemeyer, T., Wingender, E., Reuter, I., Hermjakob, H., Kel, A.E., Kolpakov, F.A., Podkolodny, N.L., Kolchanov, N.A. Databases on transcriptional regulation: TRANFAC, TRRD and COMPEL. *Nucleic Acids Res*. **26**, 364-370 (1998).
